# Refractory small cell lung cancer with pancreatic metastasis: A case report

**DOI:** 10.1097/MD.0000000000041167

**Published:** 2025-01-10

**Authors:** Zhimin Xiao, Yan Gu

**Affiliations:** a Department of Respiratory and Critical Care Medicine, The Affiliated Hospital of Inner Mongolia Medical University, Inner Mongolia, Hohhot, China.

**Keywords:** localized radiotherapy, pancreatic metastasis, refractory small cell lung cancer

## Abstract

**Rationale:**

The occurrence of refractory small cell lung cancer (rSCLC) with pancreatic metastasis is a relatively rare clinical condition, which is typically accompanied by a poor prognosis and rapid disease progression.

**Patient Concerns:**

A 65-year-old male farmer from China was diagnosed with limited-stage small cell lung cancer (SCLC) 8 months ago. Following 6 cycles of EP chemotherapy, the patient’s tumor response showed partial relief. He discontinued chemotherapy for 2 months and subsequently experienced abdominal pain for 1 month.

**Diagnoses:**

A computed tomography scan revealed a malignant tumor in the lower lobe of the right lung, as well as low vascular nodules in the pancreas, which are suspected to be metastases. Laboratory tests indicated elevated levels of amylase and lipase, along with abnormalities in 5 indicators associated with lung cancer.

**Interventions:**

Upon admission, the patient received active treatment for pancreatitis. Given the progression of the tumor, salvage chemotherapy was initiated, combining albumin with paclitaxel and attilizumab, in consultation with a multidisciplinary team (MDT).

**Outcomes:**

Abdominal pain was significantly reduced, and levels of amylase and lipase returned to normal, while neuron-specific enolase levels decreased. However, during subsequent follow-up, enlarged pancreatic lesions were identified, necessitating a reevaluation of treatment strategies.

**Lessons:**

This case study highlights the challenges associated with treating rSCLC that has metastasized to the pancreas. Although targeted therapy for metastasis-induced pancreatitis and systemic chemotherapy can alleviate symptoms and enhance patients’ quality of life, continuous monitoring and optimization of treatment plans are essential for managing this aggressive disease. The findings emphasize the importance of a multidisciplinary approach in diagnosing and treating such complex cases, underscoring the necessity for personalized treatment strategies to address both the primary cancer and its metastatic complications.

## 1. Introduction

Lung cancer is the most prevalent and lethal malignant tumor.^[1]^ Small cell lung cancer (SCLC) is a distinct subtype characterized by early metastasis, rapid growth, and poor survival rates.^[2]^ Most patients are diagnosed with distant metastasis, which commonly involves the lungs, liver, bones, brain, and adrenal glands.^[3]^ Pancreatic metastasis is relatively rare. Refractory small cell lung cancer (rSCLC) is defined as SCLC with a progression-free interval of <90 days following the completion of first-line chemotherapy.^[4]^ This article presents a case of refractory lung cancer with pancreatic metastasis, aiming to raise awareness of this condition, enhance the quality of life of the affected patients, and extend their survival time.

## 2. Case description

**Figure 1.**
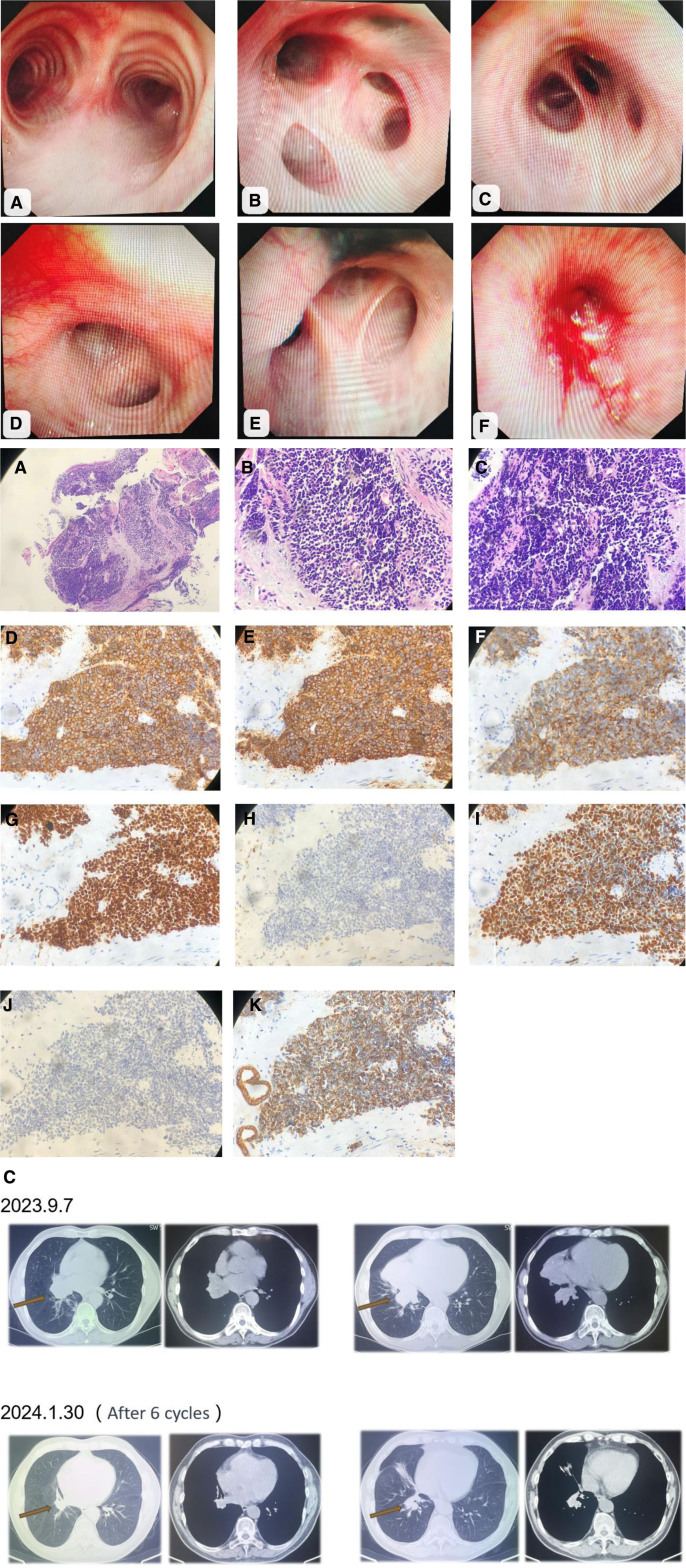
(A) Bronchoscopy examination. (B) Pathological images. (C) First-line efficacy assessment.

A 65-year-old male farmer from China was admitted on April 7, 2024, with a chief complaint of having been diagnosed with SCLC for over 8 months and experiencing abdominal pain for 1 month. The patient initially sought medical attention 8 months ago due to persistent cough and sputum production. A chest computed tomography (CT) scan revealed a mass in the right lower lung lobe accompanied by obstructive pneumonia, which was suggestive of central lung cancer and metastasis to the right pulmonary hilum and mediastinal lymph nodes. Bronchoscopy revealed a patent trachea with a sharp carina, scattered carbon deposits in the mucosa of the right upper lobe bronchus, and wide secondary carina. The right middle lobe bronchus exhibited a mass that completely obstructed the lumen, whereas all levels of the left bronchus remained patent with smooth mucosa (Fig. 1A). A biopsy of the right middle lobe bronchus confirmed a pathology consistent with small cell carcinoma of the right lung. Immunohistochemical analysis yielded the following results: CD56 (+), Syn (+), CgA (+), TTF-1 (+), LCA (–), CK (+), Ki67 (80% +), and P40 (–) (Fig. 1B). Pathology confirmed the diagnosis of SCLC, necessitating the consideration of concurrent large-cell neuroendocrine carcinoma. The first-line treatment consisted of 6 cycles of chemotherapy based on the EP regimen (etoposide + carboplatin), with treatment efficacy assessed as a partial response (PR) (Fig. 1C).

The last chemotherapy session was conducted on January 22, 2024. Approximately 1 month ago, the patient began experiencing insidious pain in the upper abdomen, which had no obvious cause and worsened with eating, lasting for 1 to 2 hours. Treatment with bismuth pectin and rabeprazole over a 2-week period yielded no improvement.

**Figure 2.**
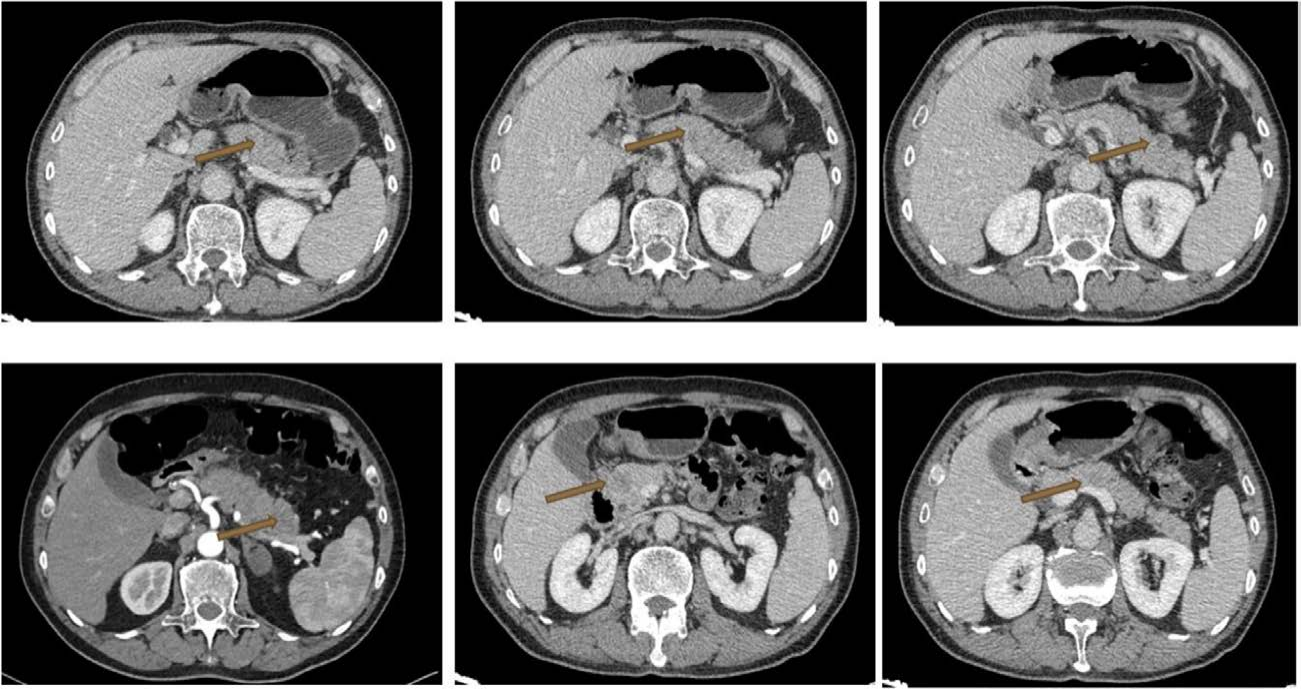
Abdominal enhanced CT at admission. CT = computer tomography.

The patient had a 20-year history of bronchial asthma. He had smoked for 10 years at an average of about 10 cigarettes per day but had been smoke-free for the past 20 years. Additionally, he consumed 100 mL of alcohol daily with a 40-year drinking history. There was no reported family history of the tumors. Upon admission, physical examination revealed a supple neck with no enlargement of superficial lymph nodes. Cardiac and pulmonary examination revealed no obvious abnormalities. The abdomen was soft and tender in the upper region. However, no rebound tenderness or masses were noted, and the remaining examination findings were unremarkable. Admission laboratory tests indicated the following: Complete blood count revealed a white blood cell count of 6.01 × 10^9^/L, a lymphocyte percentage of 8.60%, a neutrophil percentage of 77.50%, and a lymphocyte count of 0.52 × 10^9^/L. Serum amylase and lipase levels were 911.4 U/L, 229,911 U/L, and 3263 U/L, respectively. Tumor markers included carcinoembryonic antigen (CEA) at 5.07 ng/mL, neuron-specific enolase (NSE) at 39.57 ng/mL, and gastrin-releasing peptide precursor exceeding 5000.00 pg/mL. Liver function tests showed an AST/ALT ratio of 0.7, gamma-glutamyl transferase at 96.5 U/L, glutamate dehydrogenase at 6.3 U/L, and prealbumin at 14.8 mg/dL. Coagulation tests indicated an activated partial thromboplastin time of 22.70 seconds, fibrinogen level of 5.22 g/L, and D-dimer level of 1.37 μg/mL. Chest enhanced CT imaging revealed central-type lung cancer in the right lower lobe, accompanied by obstructive pneumonia in the middle and lower lobes of the right lung, which had decreased in size compared to the previous scan conducted on August 29, 2023. Abdominal enhanced CT revealed multiple hypovascular nodules in the pancreas, suggesting metastatic tumors (Fig. 2). Lymph node ultrasonography revealed a hypoechoic nodule on the pancreatic body.

Based on the clinical symptoms, medical history, imaging, pathology, and laboratory test results, the diagnosis was as follows: Malignant tumor of the right lung, specifically small cell lung cancer located in the right lower lobe, staged as T3N2M1: pulmonary metastatic malignant tumor characterized by nodules within the right lung, secondary malignant tumor of the pleura, indicating possible metastasis to nodules in the right pleura, and secondary malignant tumor of the pancreas. Acute pancreatitis. Bronchial asthma.

**Figure 3.**
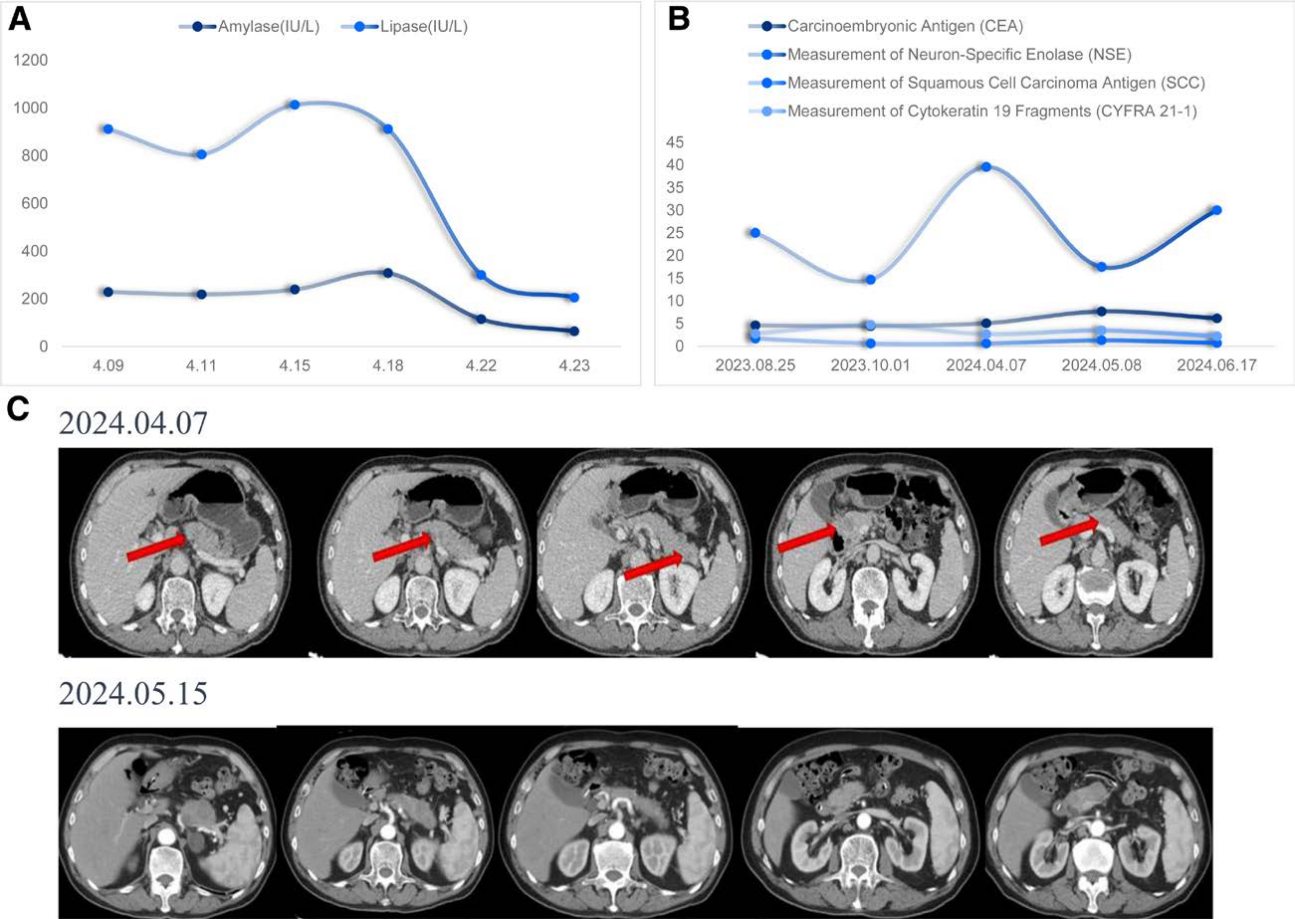
(A) Trends in enzyme changes. (B) Trends in tumor markers. (C) Follow-up abdominal CT after treatment. CT = computer tomography.

Treatment course: prior to admission, the patient underwent 6 cycles of EP chemotherapy, which resulted in a PR in treatment efficacy assessment. Between January 22 and April 7, 2024, there was a treatment hiatus during which new pancreatic lesions developed along with the progression of the pulmonary disease. Upon admission, conservative treatment was administered for 7 days, including nasoenteric nutrition, acid suppression, somatostatin, ulinastatin, gabapentin, anti-infection therapy, enemas, correction of electrolyte imbalances, and parenteral nutrition. Despite these interventions, upper abdominal pain persisted, and amylase levels exhibited a minimal decrease. Following an MDT discussion, salvage chemotherapy was initiated on April 21, 2024, consisting of albumin-bound paclitaxel at a dose of 200 mg on days 1 and 8, and atezolizumab at 1200 mg on day 4. After treatment, the patient’s abdominal pain significantly improved, amylase levels normalized (Table 1 and Fig. 3A), and NSE levels significantly decreased (Fig. 3B), indicating an effective treatment. A follow-up abdominal CT scan, however, revealed an increase in the size of the pancreatic lesions compared to the previous imaging (Fig. 3C).

**Table 1:** Changes in amylase and lipase.

	04.09	04.11	04.15	04.18	04.22	04.23
Amylase (IU/L)	229	219	239	308	115	64
Lipase (IU/L)	911.4	805.4	1013.5	911.6	300.6	205.6

## 3. Discussion

rSCLC with concurrent pancreatic metastasis is a relatively rare condition associated with a poor prognosis. Hou et al^[5]^ reported that primary tumor types metastasize to the pancreas, second most commonly to the lung, and that lung adenocarcinomas are more frequently metastatic to the pancreas than squamous cell carcinomas and SCLC. Metastasis to the pancreas. However, it has also been reported that SCLC is a common type of pathology leading to pancreatic metastasis.^[6]^ This study aimed to investigate the clinical data of a patient with refractory SCLC and pancreatic metastasis, including the diagnosis, differential diagnosis, treatment, and prognosis.

An important limitation of this study is its focus on a single patient, which restricts the generalizability of the findings. Additionally, the patient did not undergo histopathological or cytological examination, which are considered the gold standards for diagnosing pancreatic lesions.^[5,7]^ Consequently, the presence of pancreatic lesions does not rule out the possibility of primary cancer. Moreover, literature indicates that the incidence of new tumors in cancer patients is significantly higher than that in the general population.^[8]^ Multiple primary cancer refers to the occurrence of 2 or more primary malignant tumors, either simultaneously or successively.^[9]^ Given the substantial differences in origin, histological features, stage, treatment, and prognosis between multiple primary and metastatic cancers, it is crucial to determine whether pancreatic lesions are primary or metastatic for accurate diagnosis and effective treatment.

Several cases of SCLC with pancreatic metastasis, primarily presenting as acute pancreatitis, have been reported. In these instances, amylase levels decreased, and the metastatic lesions were absorbed following chemotherapy with the EP regimen.^[10]^ Additionally, Burmeister et al^[11]^ reported a similar case. These findings suggest that patients undergoing chemotherapy may experience improved survival rates. Second-line chemotherapy is commonly employed for the treatment of rSCLC. Topotecan, a topoisomerase I inhibitor, was the only drug approved by the Food and Drug Administration (FDA) in 1998 for use in recurrent or progressive SCLC. It is considered as a standard second-line chemotherapy drug in many countries.^[12,13]^ However, topotecan has limited efficacy against rSCLC with studies indicating an objective remission rate of only 5%.^[14]^ Amrubicin is a topoisomerase II inhibitor.^[15]^ Pawel et al, in their study on second-line treatment of rSCLC compared to topotecan, found that Amrubicin provided longer median overall survival (6.2 months vs 5.7 months) and progression-free survival (4.1 months vs 3.5 months). Although both groups experienced fewer adverse reactions when compared to each other, the amorubicin group had lower incidences of anemia, leukopenia, and thrombocytopenia than the topotecan group but higher incidence of febrile neutropenia. In summary, Amrubicin demonstrated better efficacy in treating rSCLC. In June 2020, lurbinectedin was approved by FDA as a second-line treatment for SCLC that progresses during or after platinum therapy.^[16]^ Both alone and in combination with doxorubicin paclitaxel or irinotecan have shown significant efficacy against rSCLC. Irinotecan is also a topoisomerase I inhibitor. As reported by Xing et al.^[17]^, the study revealed that patients undergoing second-line chemotherapy with single-drug irinotecan exhibited a median progression-free survival time of 3.8 months and a median overall survival time of 8.1 months, while experiencing minimal incidence of adverse reactions. These findings highlight the significant efficacy of irinotecan in rSCLC.

The patient presented with acute pancreatitis. After active treatment failed, we convened a MDT discussion to consider the possibility of platinum resistance and combined non-small cell carcinoma. Albumin-paclitaxel combined with atezolizumab was selected for subsequent treatment to provide survival benefits. After 1 cycle of chemotherapy, the abdominal pain was relieved, the serum amylase level normalized, and the lung lesions improved. However, the size of the pancreatic lesions increased, and the patient’s abdominal pain subsided significantly. Follow-up abdominal CT scans showed no obvious fluid accumulation around the pancreas, and the pancreas maintained its normal shape and size. After chemotherapy, the patient’s condition stabilized. We considered the observed change to be a sign of the later stages of inflammation dissipation. Subsequently, the nasojejunal feeding tube was successfully removed, and the patient’s nutritional status gradually improved with oral intake. EUSFNA was recommended for a definite diagnosis, but the family refused.

After that, we went through 2 cycles of chemotherapy (album paclitaxel 150 mg d1 and d8, atezolizumab 1200 mg d8). When the patient’s general condition was stable, we considered local radiotherapy to relieve local symptoms and improve physical condition thereby. Although radiotherapy can cause damage, low-dose radiotherapy will not cause serious adverse reactions in patients with SCLC combined with abdominal metastasis. Follow-up abdominal CT examination showed that the pancreatic metastasis lesions were smaller than before, and the patient’s general condition was good. Of note, the efficacy of these agents may vary depending on individual patient characteristics and resistance to prior therapy. Therefore, a personalized treatment plan must take into account the patient’s overall condition, treatment history, and potential adverse effects.

## 4. Conclusion

When encountering cases of acute pancreatitis in patients with refractory SCLC, we must consider the possibility of metastasis-induced acute pancreatitis, and chemotherapy combined with radiotherapy may provide substantial benefit if pancreatic metastases are detected in these patients.^[3,10]^ In conclusion, refractory small cell lung cancer with pancreatic metastasis is a rare and complex disease that requires multidisciplinary collaboration to develop personalized treatment plans. Although this single-patient study has limitations, the findings contribute to a better understanding of the disease and its treatment. Future studies with larger sample sizes and more defined diagnostic methods are needed to further elucidate optimal treatment strategies for this challenging disease.

## Acknowledgments

The authors thank the patient and his relatives for agreeing to report his case and providing a detailed medical history.

## Author contributions

**Writing – original draft:** Zhimin Xiao.

**Writing – review & editing:** Zhimin Xiao, Yan Gu.
